# A technique to treat Descemet’s membrane detachment following cataract surgery

**DOI:** 10.3389/fmed.2024.1402853

**Published:** 2024-06-11

**Authors:** Wenjie Liu, Jack X. Ma, Xin Tan, Feiyan Chai, Jiewei Liu

**Affiliations:** ^1^Department of Cataract, Shanxi Eye Hospital, Taiyuan, Shanxi, China; ^2^Department of Ophthalmology, Baylor College of Medicine, Houston, TX, United States

**Keywords:** Descemet’s membrane detachment, cataract surgery, reattachment of Descemet's membrane detachment, DMD, cataract

## Abstract

We describe a technique to reattach the detached Descemet’s membrane, following cataract surgery. From the main clear corneal cataract incision, aqueous humor is ejected completely by apposition of the cornea to the iris for approximately 3 s. This ensures the fluid in the space between the stroma and Descemet’s membrane is ejected and the detached Descemet’s membrane returns to its original position. Sterile air is injected through a paracentesis 180 degrees away from the Descemet’s membrane detachment, to maintain a complete air-filled chamber. Full air tamponade is maintained for 20 min, following which one-third of the air is ejected from the chamber to prevent an increase of postoperative intraocular pressure.

## Introduction

Descemet’s membrane is the basement membrane for the corneal endothelium. Descemet’s membrane and corneal endothelium play a pivotal role in maintaining corneal clarity. Descemet’s membrane detachment (DMD) is a well-recognized and potential vision-threatening complication following cataract surgery (1). Corneal edema appears over the area of DMD and bullous keratopathy may occur, if not managed appropriately. Prompt reattachment of Descemet’s membrane may restore corneal clarity immediately and prevent wrinkling, scrolling, fibrosis, and scarring of Descemet’s membrane (2).

Conservative management is used as an approach to treat mild and nonscrolled DMD (3–7). Spontaneous resolution of corneal edema and reattachment of DMD in early and late cases has been reported. However, early surgical treatment is now advocated for cases with severe, scrolled, extensive, and visually impairing DMDs (1–9).

Pneumodescemetopexy with intracameral injection of air or gases has become the preferred treatment for DMD. Due to the concerns of endothelial toxicity induced by gases (10), there is a trend toward using intracameral air alone in repairing DMD (9). In this report, we present a technique to manage DMD following cataract surgery.

## Surgical technique

**Figure 1:**
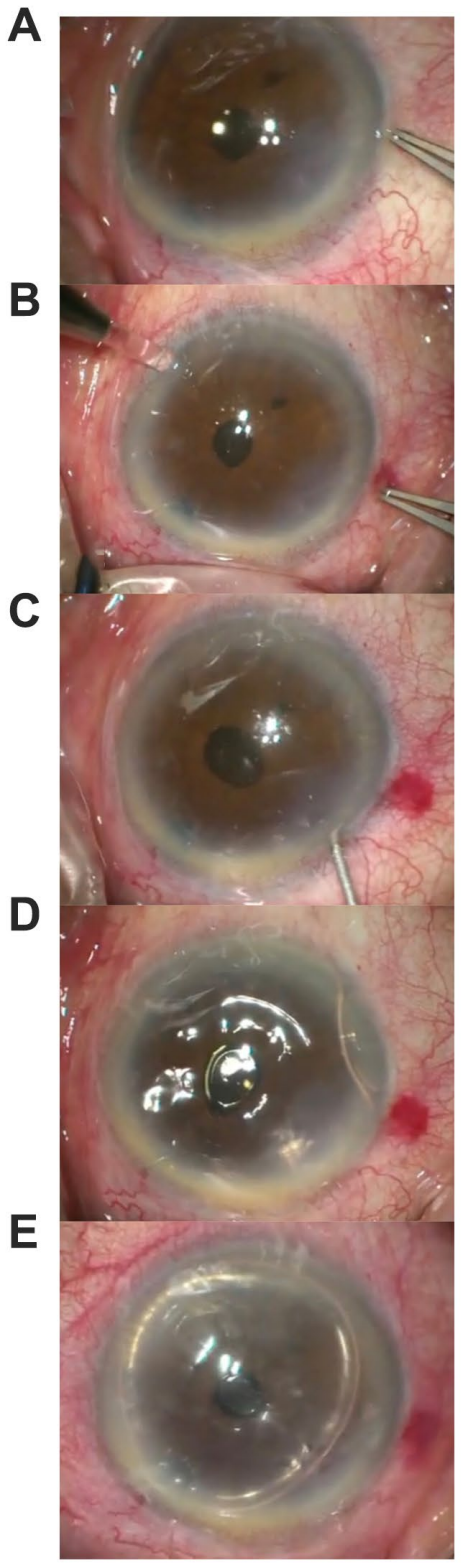
Images showing steps of surgical steps: **(A)** shows DMD; **(B)** shows a paracentesis is made at the opposite site of the DMD; **(C)** shows aqueous humor is ejected from the main clear corneal cataract incision, the cornea is briefly apposed to the iris; **(D)** shows sterile air is injected into the anterior chamber to maintain a complete air-filled chamber; **(E)** shows approximately one third of the air is ejected from the chamber after 20 min.

This technique has four steps. First, a paracentesis is made on the opposite side of the DMD. Second, the aqueous humor is ejected from the main clear corneal cataract incision. To ensure complete ejection of aqueous humor from the anterior chamber, the cornea is apposed to the iris for a short time duration. This approach squeezes out the fluid in the space between the stroma and Descemet’s membrane and ensures the detached Descemet’s membrane returns to its original position. To prevent damage to the corneal endothelial cells, apposition of the cornea to the iris is limited to a short duration of 3 s. Third, from the paracentesis, sterile air is injected into the anterior chamber to maintain a complete air-filled chamber and sustained air tamponade. Finally, full-air tamponade is maintained for 20 min, and then approximately one-third of the air is ejected from the chamber to prevent an increase of intraocular pressure (IOP) after the procedure (Figure 1).

## Results

**Figure 2:**
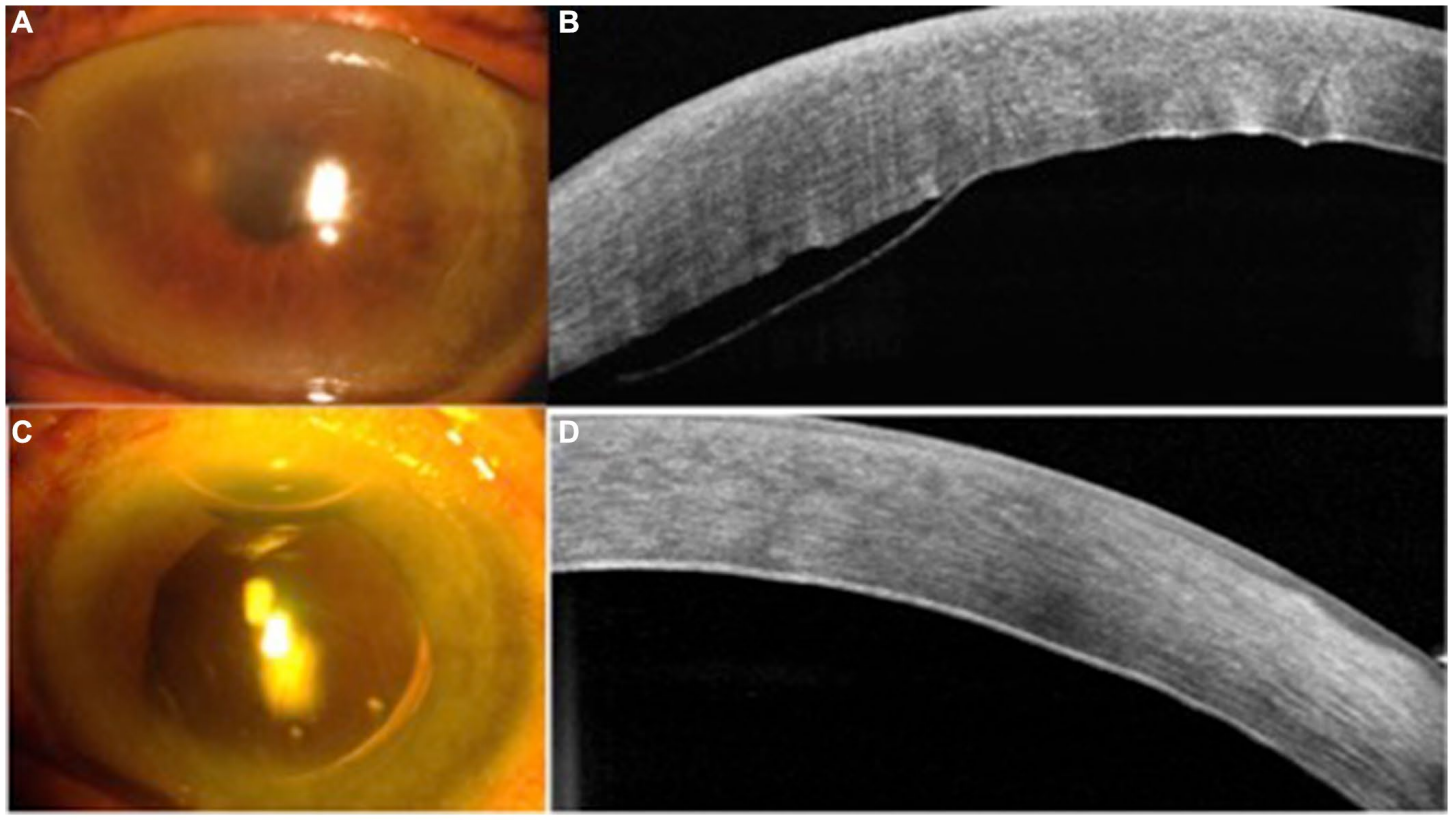
Case #5 slit lamp photos and anterior segment optical coherence tomography (OCT) maps. **(A)** corneal edema at 1 day after the cataract surgery; **(B)** Descemet’s membrane detachment (DMD) seen on OCT map; **(C)** cornea clear at 1 day following the DMD reattachment procedure; and **(D)** DMD reattached.

Five cases with DMD and reattachment procedures are listed in Table 1. From among these five, two cases, one with air injection alone into the anterior chamber (case #1) and second with complete ejection of aqueous humor without air injection (case #3) failed to reattach the Descemet’s membrane. Complete aqueous ejection followed by air injection increased IOP (cases #1 and #2). In cases 4 and 5, complete aqueous ejection, full air tamponade, and ejection of one-third of air successfully reattached the DMD (Figure 2).

**Table 1:** Cases with Descemet’s membrane detachment (DMD) and reattachment procedures.

	Case #1	Case #2	Case #3	Case #4	Case #5
Sex	Female	Male	Male	Female	Female
Age	75	88	82	80	66
Eye	Left eye	Left eye	Right eye	Right eye	Right eye
DMD occurred	Intraoperative	POD 1	Intraoperative	POD 1	POD 1
Time of reattachment procedure	End of surgery	POD 1	End of surgery	POD 12	POD 1
Reattachment procedure	Air injection	Cornea and iris apposition + air injection	Cornea and iris apposition	Cornea and iris apposition + air injection +1/3 air ejection	Cornea and iris apposition + air injection +1/3 air ejection
Results	DMD reattached	DMD reattached, high IOP	DMD reattachment failed	DMD reattached	DMD reattached
Second DMD occurred	POD 5	no	POD 1	no	no
Reattachment procedure	Cornea and iris apposition + air injection		Cornea and iris apposition + air injection +1/3 air ejection		
Results	DMD reattached, high IOP		DMD reattached		
CECC before cataract surgery	-	-	-	1357.0/mm^2^	2290.4/mm^2^
CECC before DMD reattachment procedure	-	-	-	1239.9/mm^2^	-
CECC after DMD reattachment procedure	-	-	-	1194.2/mm^2^ on day 1, and 1171.9/mm^2^ on day 6	2,139/mm^2^ on day 1, and 2279.8/mm^2^ at 1 month

POD, postoperative day; IOP, intraocular pressure; CECC, corneal endothelial cell count with the specular microscope SP-3000P (Topcon Medical Systems, Inc., Oakland, NJ); −, not available.

## Discussion

Air injection into the anterior chamber is the most common procedure used to treat DMD. Unsuccessful pneumatic descemetopexy occurs when there is fluid entrapped in the supra-descemet’s space, which prevents the apposition of Descemet’s membrane to the stroma. In such cases, an alternate method of fluid drainage by corneal venting incision or external stab incision, with or without air tamponade, has been reported (10). Internal aspiration with a needle may also be used for drainage of the fluid in the supra-descemet’s space, but these methods are complicated and prone to cause corneal damage.

Our procedure is easy to perform. By apposition of the cornea to the iris, the underlying principle of our technique is to: (1) squeeze out the fluid in the supra-descemet’s space to ensure that the Descemet’s membrane returns to its anatomical position, and (2) completely eject aqueous humor out of the anterior chamber so that the anterior chamber can be completely filled with air to ensure effective air tamponade. In case #1, sterile air was injected into the anterior chamber at the end of the surgery without complete ejection of the fluid in the anterior chamber anda complete air-fill was achieved in the anterior chamber. Detached Descemet’s membrane was reattached to the cornea at the end of surgery, but detachment was noted again on the 5th Post-operative Day (POD 5). Air injection into the anterior chamber is another important step. In case #3, although we completely squeezed out aqueous humor and ensured the apposition of the cornea to the iris without air injection at the end of the procedure, Descemet’s membrane detached again on POD 1. This indicates that it is important to maintain full-air tamponade for 20 min. In case #1 and case #2, both without air ejection after the surgery, the IOP increased on POD 1. To prevent this from happening, approximately one-third of the air is ejected from the chamber after full air tamponade is maintained for 20 min. In our last two cases, the IOP did not increase postoperatively.

Prolonged apposition of the iris to angle structures of the eye can cause permanent peripheral anterior synechiae and chronic angle-closure glaucoma. Corneal contact with vitreous humor or an IOL (Intraocular Lens implant) can result in endothelial cell loss and chronic corneal edema. With our technique, the cornea touches the iris for only a few seconds, which should not cause peripheral anterior synechiae or corneal endothelial cell damage. Due to diffuse corneal edema, the Corneal Endothelial Cell Count (CECC) before the DMD reattachment procedure could not be obtained in cases #1–3. In case #4, CECC decreased by 3.7% on day 1 and 5.5% on day 6 following the DMD reattachment procedure. In this case, the CECC before the cataract surgery was low, presumably caused by previous glaucoma surgery. In case #5, the CECC at 1 month after the DMD reattachment procedure was similar to the CECC before the cataract surgery. Long-term follow-up on CECC is needed in cases undergoing DMD reattachment procedures.

Kumar et al. (1) introduced an algorithm that classifies Descemet membrane detachment (DMD) based on anterior segment optical coherence tomography (AS-OCT) imaging parameters. The classification is made according to the height and length of the detachment, as well as the area affected and whether it involves the pupil. Specifically, they categorized DMD into three groups based on height: under 100 micrometers, between 100 and 300 micrometers, and over 300 micrometers. The length was similarly divided into three categories: under 1 millimeter, between 1 and 2 millimeters, and over 2 millimeters. The extent and pupil involvement were divided into zones: zone 1 for the central 5 mm, zone 2 for the paracentral 5-8 mm, and zone 3 for the periphery beyond 8 mm. The eyes were then allocated to medical or surgical treatment, with their outcomes assessed in terms of functionality and anatomy.

Mackool and Holtz (11) further refined the understanding of DMD after surgery. They distinguished between planar and non-planar DMD based on the degree of separation in the detached membrane. A planar DMD, with less than 1 mm of separation, typically reattaches on its own and has a more favorable prognosis compared to non-planar DMD, which has a separation greater than 1 mm and usually requires surgical management. Additionally, they identified a peripheral type of DMD located within the 3 mm range from the limbus, which can be monitored without immediate intervention and a combined central and peripheral DMD that necessitates treatment.

In managing DMD, it’s crucial to emphasize that prevention measures are more effective than treatment post-facto. A preoperatively low endothelial cell count has been identified as a substantial risk indicator; thus, it is advisable to proceed with heightened vigilance in such instances (7). When performing the primary surgical incision and creating access ports, disposable sharp blades are recommended for their precision and to minimize trauma to surrounding tissue. Furthermore, special care must be taken when inserting cannulas for delivering viscoelastic material and hydrating the corneal incisions with a balanced salt solution (BSS).

Early intervention could be instrumental in preventing complications associated with prolonged DMD. Delaying treatment might result in an excessive loss of endothelial cells, potentially leading to bullous keratopathy. Our technology offers a safe and effective approach for treating early-stage DMD before it progresses to more advanced stages that might require complex procedures such as penetrating keratoplasty, Descemet’s Stripping Endothelial Keratoplasty (DSEK), or Descemet Membrane (DM) endothelial keratoplasty.

Our technique is advocated for early cases of DMD. DMD following cataract surgery produces corneal edema. Early surgical treatment should be performed to prevent decreased vision. If the detached Descemet’s membrane cannot be reattached with conservative management, the detached Descemet’s membrane may become stiff or scrolled, and surgical management may become difficult. If extensive DMD is observed after the surgery, the reattachment procedure should be performed immediately.

In summary, our case samples demonstrate that this technique is a simple and effective approach to managing DMD following cataract surgery. Further study is needed to evaluate its long-term safety in a large number of cases.

## Data availability statement

The original contributions presented in the study are included in the article/supplementary material, further inquiries can be directed to the corresponding author.

## Ethics statement

The studies involving humans were approved by Ethics Committee of Shanxi Eye Hospital. The studies were conducted in accordance with the local legislation and institutional requirements. Written informed consent for participation was not required from the participants or the participants’ legal guardians/next of kin in accordance with the national legislation and institutional requirements. Written informed consent was obtained from the minor(s)’ legal guardian/next of kin for the publication of any potentially identifiable images or data included in this article.

## Author contributions

WL: Writing – original draft. JM: Writing – review & editing. XT: Writing – review & editing. FC: Writing – review & editing. JL: Writing – review & editing.
